# Bauerenol Acetate, the Pentacyclic Triterpenoid from *Tabernaemontana longipes*, is an Antitrypanosomal Agent

**DOI:** 10.3390/molecules23020355

**Published:** 2018-02-08

**Authors:** Simira Carothers, Rogers Nyamwihura, Jasmine Collins, Huaisheng Zhang, HaJeung Park, William N. Setzer, Ifedayo Victor Ogungbe

**Affiliations:** 1 Department of Chemistry, Jackson State University, Jackson, MS 39217, USA; Simira.carothers@gmail.com (S.C.); rogersnyamwihura@yahoo.com (R.N.); Jcollins1908@gmail.com (J.C.); huiasheng.zhang@students.jsums.edu (H.Z.); 2 X-ray Crystallography Laboratory, Scripps Research Institute-FL, Jupiter, FL 33458, USA; hajpark@scripps.edu; 3 Department of Chemistry, University of Alabama in Huntsville, Huntsville, AL 35899, USA; wsetzer@chemistry.uah.edu

**Keywords:** *Tabernaemontana longipes*, *Trypanosoma brucei*, trypanosomiasis, pentacyclic triterpenoid

## Abstract

The Latin American plant *Tabernaemontana longipes* was studied in this work as a potential source of antiparasitic agents. The chloroform extract of *T. longipes* leaves was separated into several fractions, and tested for antitrypanosomal activity. One of the fractions displayed significant growth inhibitory activity against *Trypanosoma brucei*. The active principle in the fraction was isolated, purified, and characterized by NMR and mass spectrometry. The antitrypanosomal agent in the CHCl_3_ extract of *T. longipes* leaves is the pentacyclic triterpenoid bauerenol acetate. A metabolite profiling assay suggest that the triterpenoid influences cholesterol metabolism. The molecular target(s) of bauerenol and its acetate, like many other antiparasitic pentacyclic triterpenoids is/are unknown, but they present privileged structural scaffolds that can be explored for structure-based activity optimization studies using phenotypic assays.

## 1. Introduction

African sleeping sickness (Human African Trypanosomiasis) caused by protozoan *Trypanosoma brucei gambiense/rhodesiense* is still a public health problem in rural and remote regions of the continent, especially in the Democratic Republic of the Congo. Despite a gradual decrease in the number of reported cases in the past few years, the lack of effective and safe medicines, the unavailability of adequate and rapid diagnostic tools, especially in rural and remote places, and the possibility of continuous transmission of the parasite from animal reservoirs to humans, make the disease a continuous threat to millions of people [1,2,3,4]. To consolidate recent gains in the fight against the disease, continued integration of discovery and development of new drugs and new diagnostic tools with disease control and prevention strategies is of paramount importance. Therefore, discovery of new antitrypanosomal chemical entities using molecular target-based or phenotypic screening-based approaches remains very attractive.

In our on-going exploration of tropical plants as potential sources of new antitrypanosomal agents, *Tabernaemontana longipes* Donn. Sm. (Apocynaceae) was studied as a potential source of antitrypanosomal phytochemicals. *T. longipes* is a tropical plant found in several Latin American countries, including Nicaragua, Colombia, Ecuador, and Costa Rica. The plant’s flowers are white or cream-colored, with cylindrical corollas and brown lobes, and the flowers smell sweet [5,6].

## 2. Results and Discussion

**Figure 1:**
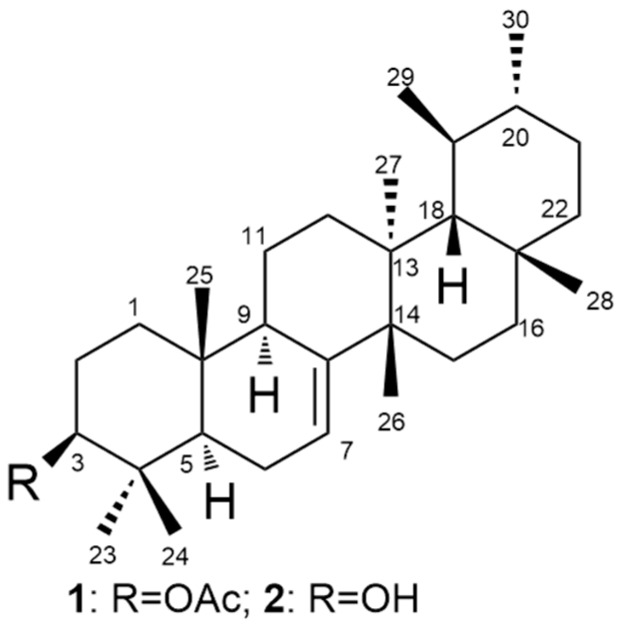
Structure of compounds **1** and **2**.

The leaves of *T. longipes* were obtained from several mature trees growing in Costa Rica in 2009. The leaves (1 kg) were dried, chopped, and extracted with refluxing chloroform for 6 h. The chloroform extract (14 g) was fractionated on silica gel (700 g) and eluted with 5:1 hexane/ethyl acetate mixture to obtain 49 fractions. The fractions were pooled together to obtain nine superfractions (SP1–9). The fractions were screened for their ability to inhibit the growth of *T. brucei* in vitro. SP2 displayed significant antitrypanosomal activity (76% growth inhibition at 10 µg/mL). SP2 (2.1 g) was subsequently separated using 10:1 hexane/ethyl acetate mixture over 200 g of silica gel to obtain seven fractions (SP2F1–SP2F7). The TLC profiles of the fractions showed that SP2F4 and SP2F5 have a major and similar component while the other fractions have several minor components. SP2F4 and SP2F5 were pooled together and purified by crystallization. SP2F4-5 (227 mg), a yellowish, oily amorphous solid, was dissolved in hexane and placed in a pentane tank for 24 h to induce crystallization. The resulting crystals were filtered, washed four times with pentane (2 mL), and allowed to dry, to give colorless needle-like crystals (1, 76 mg). The ^1^H- and ^13^C-NMR spectra of 1 suggested that it is a triterpenoid. The ^13^C-NMR spectrum shows 31 distinguishable carbon signals. The most characteristic structural features in the ^13^C spectrum include an ester carbonyl signal (δ 171.1) and an alkenyl carbon signal (δ 145.6). The ^1^H and HSQC spectra revealed that there are nine methyl groups, nine methylene groups, one alkenyl hydrogen, one oxygenated methine group, five non-oxygenated methine groups, and five quaternary carbons (Table 1 and Figure 1).

The connectivity of the carbon-carbon atoms, and their hydrogen atoms was deciphered using a combination of HMBC, COSY, and HSQC data (Table 1 and Figure S1 in Supplementary File 1). The HMBC data provide a clear correlation between one of the methyl groups and the carbonyl carbon that is typical of acetylated triterpenoid, while the carbon connectivity suggested that is a pentacyclic triterpenoid [7]. The relative stereochemistry of the compound was established by nuclear Overhauser effect (NOE) correlations, and X-ray diffraction data from a single plate crystal.

Its molecular formula, C_32_H_52_O_2_, was established from high resolution mass spectrometry (HRMS; TOFMS ESI+, [M + Li]^+^ *m*/*z* 475.4113, for C_32_H_52_O_2_Li, Figure S2), and CH elemental analysis (Found: C = 81.37% and H = 11.13%; Calculated: C = 81.99% and H = 11.18%). The compound (**1**) was identified as bauerenol acetate, a pentacyclic triterpenoid that had been previously identified in *Tabernaemontana longipes* [8,9]. Compound **1** was then tested for its ability to inhibit the growth of *T. brucei* in vitro. The results showed that bauerenol acetate has an IC_50_ value of 3.1 µM (Table 2).

Due to the relatively poor solubility of the acetate in DMSO during the bioassay, the acetate was subjected to hydrolysis in 5% ethanolic KOH to obtain its alcohol derivative bauerenol (**2**). The solubility of bauerenol in DMSO was somewhat better than the acetate, and its activity on *T. brucei* in vitro was characterized by an IC_50_ value of 2.7 µM. The antitrypanosomal activities of these compounds are much lower (1–3 order of magnitude) than clinically used antitrypanosomal drugs [10]. Also, the IC_50_ values of **1** and **2** are higher than the IC_50_ value (<1 µM) proposed by Nwaka and Hudson as criteria for preclinical evaluation of antitrypanosomal drug candidates [11]. A number of oleanolic and ursolic acid-based pentacyclic triterpenoids have previously reported to have growth inhibitory activity against *Leishmania* spp. and *T. cruzi* but not *T. brucei* [12,13,14,15,16,17].

Several pentacyclic triterpenoids have been shown to be cytotoxic or described as potential anticancer agents [18,19,20]. Therefore, we decided to test if **1** and **2** are cytotoxic to human hepatocarcinoma cell model (Hep G2) or if they show selective antitrypanosomal activity. Both compounds have very little effect on cellular viability on Hep G2 cells (IC_50_ > 80 µM). The molecular target (macromolecule or pathway) of these compounds is yet to be experimentally determined and could be the subject of future studies, but previous docking studies have suggested *T. brucei* sterol 14α-demethylase to be a potential target for triterpenoids [21]. 

**Figure 2:**
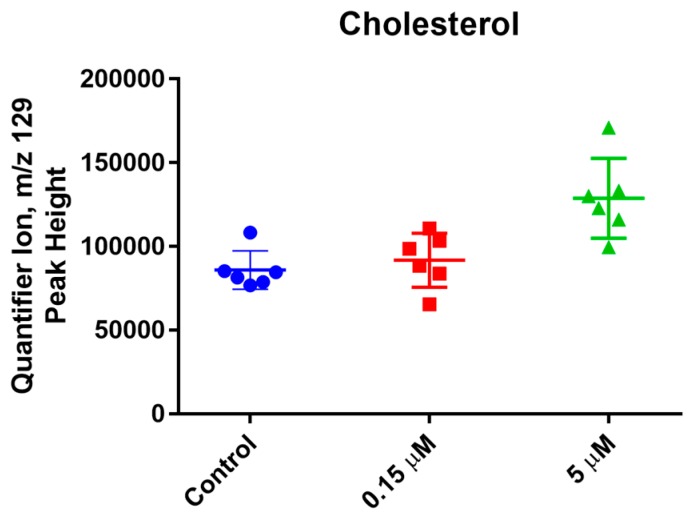
Levels of cholesterol in parasite treated with bauerenol (**2**). The levels of cholesterol in the parasite was significantly increased after 4-h exposure to 5 µM of **2**.

Nevertheless, to understand how the pentacyclic triterpenoid (**2**) affect *T. brucei* cells, a primary metabolite profiling assay was carried out. The metabolites were identified and quantified using GC-TOFMS as outlined in the methods section below. A total of 90 metabolites were unambiguously identified and quantified (Supplementary File 2) in the assay. The metabolites with the most notable concentration-dependent changes in the parasites are stearic acid, phosphoenolpyruvate, myristic acid, cholesterol, arachidic acid, and adenine. The changes in the levels of cholesterol were the most consistent and conceivably the most informative from the metabolite profiling assay. The levels of cholesterol (Figure 2 and Figure S3) in parasites treated with 5 µM of **2** was significantly increased when compared to the control group (*n* = 6, *p*-value = 0.0023) and the group treated with 0.15 µM of **2** (*n* = 6, *p*-value = 0.0072). This suggests that the triterpenoid is interfering with the metabolism of endogenous sterol(s) or a cholesterol-dependent pathway. Previous studies have shown that accumulation of cholesterol by trypanosomes is associated with perturbation of the endogenous sterol biosynthetic pathway [22,23,24,25]. Therefore, sterol biosynthetic pathway in *T. brucei* is the likely target of bauerenol (**2**). 

In conclusion, bauerenol and its acetate are anti-*T. brucei* agents. The structure of bauerenol (**2**) can be used as a starting structural motif in natural products-based diversity oriented synthesis (DOS) or in the synthesis of derivatives that have better drug-like physicochemical properties. Further studies are necessary to determine the specific enzyme or metabolic step directly affected by bauerenol. 

## 3. Materials and Methods

### 3.1. Plant Material 

*T. longipes* leaves were collected in May 2009, in the Monteverde region of northwestern Costa Rica (10°18′3.4′′ N, 84°48′39.3′′ W, 1381 m elevation). The plant was identified by William A. Haber. A voucher specimen has been deposited with the Missouri Botanical Garden herbarium (Haber Collection No. 7104).

### 3.2. Compound Characterization

All chromatographic separations were carried out on Sortech’s silica gel (Premium Rf, 60 Å, 40–75 µm). The 1D (^1^H and ^13^C) and 2D (gCOSY, gHSQC, gHMBC, NOESY) data were collected on a 500 MHz (13C: 125 MHz) Varian INOVA spectrometer operated with *Vnmrj*. Chloroform-d was used as solvent, and tetramethylsilane (TMS) as internal standard in the solvent. The mass spectra were obtained on a Synapt G2 HDMS instrument (Waters; Milford, MA, USA) operated in positive ESI mode. Elemental analysis was carried out on a Costech’s ECS 4010 (Valencia, CA, USA). The infrared spectra were obtained on a Perkin Elmer Spectrum 100 FT-IR spectrometer (Waltham, MA, USA) using an ATR accessory. Please see Figures S1, S2 and S4–S7 in Supplementary File 1 for NMR, IR, Anal, and MS spectra as well as the crystal structure.

Crystals of **1** were obtained by slow evaporation of a chloroform solution of **1**. X-ray diffraction data were collected on a Bruker AXS Smart APEX CCD II [Mo Kα (λ = 0.71073 Å)] (Madison, WI, USA) at 295 K. The structure was solved by Olex2 using SHELXTL and was refined by full-matrix least-squares procedure (Figure S6) Non-hydrogen atoms were refined anisotropically. Hydrogen atoms were added in calculated positions and were refined. Melting points were recorded on a MEL-TEMP 1101D apparatus (Dubuque, IA, USA). The melting point values are uncorrected. 

#### 3.2.1. Bauerenol Acetate (**1**) 

Colorless crystals; melting point 289–291 °C; IR (CHCl_3_) ν_max_ 2935, 1732, 1463, 1374, 1249, 1026 cm^−1^; ^1^H- and ^13^C-NMR, see Table 1; HRMS ESI+ [(M + Li)^+^], *m*/*z* 475.4113 [Calculated for C_32_H_52_O_2_Li 475.4128]. 

#### 3.2.2. Bauerenol (**2**)

Colorless solid; melting point 202–203 °C; IR (CHCl_3_) ν_max_ 3379, 2929, 2866, 1457, 1372, 1032 cm^−1^; ^1^H- and ^13^C-NMR, see Table 1; HRMS ESI+ [(M + H)^+^], *m*/*z* 427.3922 [Calculated for C_30_H_51_O_2_ 427.3940].

### 3.3. Trypanosoma brucei Assay

The growth inhibitory activity of the compounds was evaluated using the Alamar blue assay [26]. Bloodstream form of *T. brucei* (strain 427) cultured in HMI-9-medium supplemented with 10% FBS, 10% Serum plus (SAFC), 0.05 mM bathocuproine sulfonate, 1.5 mM l-cysteine, 1 mM hypoxanthine, 0.2 mM β-mercaptoethanol, 0.16 mM thymidine, 1 mM pyruvate, and 0.0125% Tween 80 was used. Parasites were dispensed into a sterile 96-well plate at 5 × 10^3^ cells/well and treated with compound **1** and **2** for 24 h. The compounds were prepared in DMSO containing 1.25% Tween 80. Final concentration of Tween 80 in the assay wells was 0.0125%. The compounds were screened in triplicate with a total assay volume of 100 µL. Next, Alamar blue (20 µL) was added and the plate was incubated at 37 °C for 4 h. Immediately following incubation, fluorescence signals were read (λ_ex_ 530 nm, λ_em_ 590 nm). Suramin was used as positive control. 

### 3.4. Cytotoxicity Assay

Human hepatocarcinoma cell line (Hep G2 CRL-11997™, ATCC) was used for cytotoxicity studies. The cells were grown in complete medium (DMEM: F12 containing l-glutamine and sodium bicarbonate, 10% FBS, 1% penicillin/streptomycin, 0.0125% Tween 80) incubated at 37 °C in a 5% CO_2_ environment. Once 80–90% confluent, the cells were washed with phosphate-buffered saline (PBS), treated with 0.25% (*w*/*v*) of trypsin/EDTA, counted and suspended in fresh complete media. Into 96-well plates, 5 × 10^5^ cells/mL were seeded and incubated for 24 h. Cells were treated with the compounds prepared in DMSO containing 1.25% Tween 80 for 72 h. Final concentration of Tween 80 in the assay wells was 0.0125%. Thereafter, DMEM:F12 medium containing MTT (5 mg/mL in PBS) was added to the cells and incubated for 1 h. The MTT-containing medium was then gently removed and replaced with DMSO (200 μL/well), the plate was then mixed gently to allow the formazan crystals to dissolve. Absorbance was measured at 550 nm. All compounds were tested in triplicates. SDS (10%) was used as positive control.

### 3.5. Metabolic Profiling

*T. brucei* cells in supplemented HMI-9 medium were treated with 5 µM and 0.15 µM of **2** for 4 h. After treatment, the cells were washed three times with PBS, and the cell pellets were stored at −80 °C for subsequent GC-MS analysis. The treatment was carried in sextuplicate, including six no treatment controls. Ten million cells per replicate were used. Metabolites were extracted from the cells by adding 0.5 mL of 3:3:2 acetonitrile:isopropanol:water mixture to each sample followed by the addition of 3 stainless steel grinding beads and then transferred to a GenoGrinder (Metuchen, NJ, USA) for grinding. The cells were grinded for 30 s at 1500 rpm, and centrifuged at 14,000× *g* for 5 min. The supernatants were transferred to new tubes. The extraction was repeated twice, and the pooled supernatant was concentrated to 500 µL. The samples were silylated using MSTFA + 1% TMCS at 37 °C, and were used for primary metabolite profiling on a LECO Pegasus IV GC-time of flight mass spectrometer (Saint Joseph, MI, USA) as described by Fiehn and co-workers [25]. The GC-TOFMS analysis was carried out at the NIH West Coast Metabolomics Center at the University of California, Davis. 

The samples (0.5 µL each) were analyzed using the splitless mode on a Rtx-5Sil MS capillary column (30 m length × 0.25 mm internal diameter with 0.25 μm film made of 95% dimethyl/5% diphenylpolysiloxane, Restek Corporation, Bellefonte, PA, USA). The gas flow-rate was 1 mL/min. Initial injection temperature was set at 50 °C and ramped up to 250 °C at a rate of 12 °C/s. Initial oven temperature was set at 50 °C for 1 min, then ramped up to 330 °C at a rate of 20 °C/min, and held constant for 5 min. The TOF mass spectrometer (LECO; Saint Joseph, MI, USA) unit mass resolution was set at 17 spectra/s from 80–500 Da at −70 eV ionization energy and 1800 V detector voltage. The transfer line and the ion source temperatures were set at 230 °C and 250 °C, respectively. The primary metabolites were identified and quantified using the quantifier ions shown in Supplementary File 2. The raw mass spectra for the metabolites were processed by BinBase (BB) software (FiehnLab; Davis, CA, USA). The relative quantification was carried out using the peak height of each quantifier ion. The peak heights were normalized by dividing them by the sum of all peak heights for all identified metabolites and multiplied by the average peak heights for all identified metabolites in each sample [27,28].

## Figures and Tables

**Table 1:** ^1^H- and ^13^C-NMR Spectra Data for **1** and **2**.

	1		2	
Position	^13^C	^1^H	^13^C	^1^H
1	36.6	1.18, 1.66 (2H, m)	37.02	1.09, 1.64 (2H, m)
2	24.3	1.66 (2H, m)	27.8	1.61 (2H, m)
3	81.3	4.51 (1H, dd)	79.4	3.24 (1H, dd)
4	37.9		39.0	
5	48.3	2.21 (1H, m)	48.3	2.19 (1H, m)
6	24.1	1.97, 2.14 (2H, m)	24.3	1.97, 2.16 (2H, m)
7	116.4	5.40 (1H m)	116.5	5.41 (1H m)
8	145.6		145.4	
9	50.7	1.41 (1H, m)	50.5	1.29 (1H, m)
10	35.0		35.3	
11	32.5	1.48, 1.56 (2H, m)	32.5	1.48, 1.54 (2H, m)
12	31.6	1.14, 1.61 (2H, m)	31.6	1.14, 1.60 (2H, m)
13	37.8		37.86	
14	37.9		37.86	
15	28.8	1.40, 1.50 (2H, m)	29.0	1.41, 1.49 (2H, m)
16	16.7	1.48, 1.54 (2H, m)	16.9	1.48, 1.54 (2H, m)
17	41.2		41.3	
18	55.0	1.29 (1H, s)	55.0	1.29 (1H, s)
19	35.3	1.15 (1H, m)	35.4	1.14 (1H, m)
20	31.8	1.54 (1H, m)	31.8	1.54 (1H, m)
21	29.3	1.19, 1.51 (2H, m)	29.3	1.19, 1.51 (2H, m)
22	37.8	1.18, 1.48 (2H, m)	37.82	1.18, 1.49 (2H, m)
23	15.9	0.93 (3H, s)	14.8	0.85 (3H, s)
24	27.6	0.85 (3H, s)	27.6	0.96 (3H, s)
25	13.1	0.76 (3H, s)	13.1	0.74 (3H, s)
26	23.6	0.99 (3H, s)	23.8	0.99 (3H, s)
27	22.8	0.94 (3H, s)	22.8	0.94 (3H, s)
28	37.9	1.02 (3H, s)	38.1	1.02 (3H, 3)
29	25.8	1.04 (3H, d)	25.8	1.04 (3H, d)
30	22.6	0.90 (3H, d)	22.7	0.90 (3H, d)
OAc	21.4, 171.1	2.05 (3H, s)		

**Table 2:** Bioassay data of compounds **1** and **2**.

Compound	*T. brucei* Growth Inhibition IC_50_ (µM)	Cytotoxicity Hep G2 IC_50_ (µM)
**1**	3.09 ± 0.80	>80
**2**	2.71 ± 0.96	>80
Suramin	0.04 ± 0.01	n/a

## Supplementary Materials

The NMR, IR, CH elemental analysis, and MS spectra as well as the GC-TOFMS Data are provided as supplementary material. Supplementary materials are available online.
