# Supplementary material for: Bauerenol Acetate, the Pentacyclic Triterpenoid from Tabernaemontana longipes, is an Antitrypanosomal Agent
Source: Molecules. 2018 Feb 8;23(2):355. doi: 10.3390/molecules23020355 (PMC5911922; doi:10.3390/molecules23020355)
Supplement: Supplementary file 1 [file molecules-23-00355-s001.zip › Carothers et al_Supplementary File 1.docx]

**Supplementary Information**

**Bauerenol acetate, the pentacyclic triterpenoid from *Tabernaemontana longipes* is an antitrypanosomal agent.**

Simira Carothers^1^, Rogers Nyamwihura^1^, Jasmine Collins^1^, Huaisheng Zhang^1^, HaJeung Park^2^, William N. Setzer^3^, and Ifedayo Victor Ogungbe^1*^

^1^Department of Chemistry, Jackson State University, Jackson, MS, 39217, USA

^2^X-ray Crystallography Laboratory, Scripps Research Institute-FL, Jupiter, FL, 33458, USA

^3^Department of Chemistry, University of Alabama in Huntsville, Huntsville, AL, 35899, USA

*Email: [ifedayo.v.ogungbe@jsums.edu](mailto:ifedayo.v.ogungbe@jsums.edu)


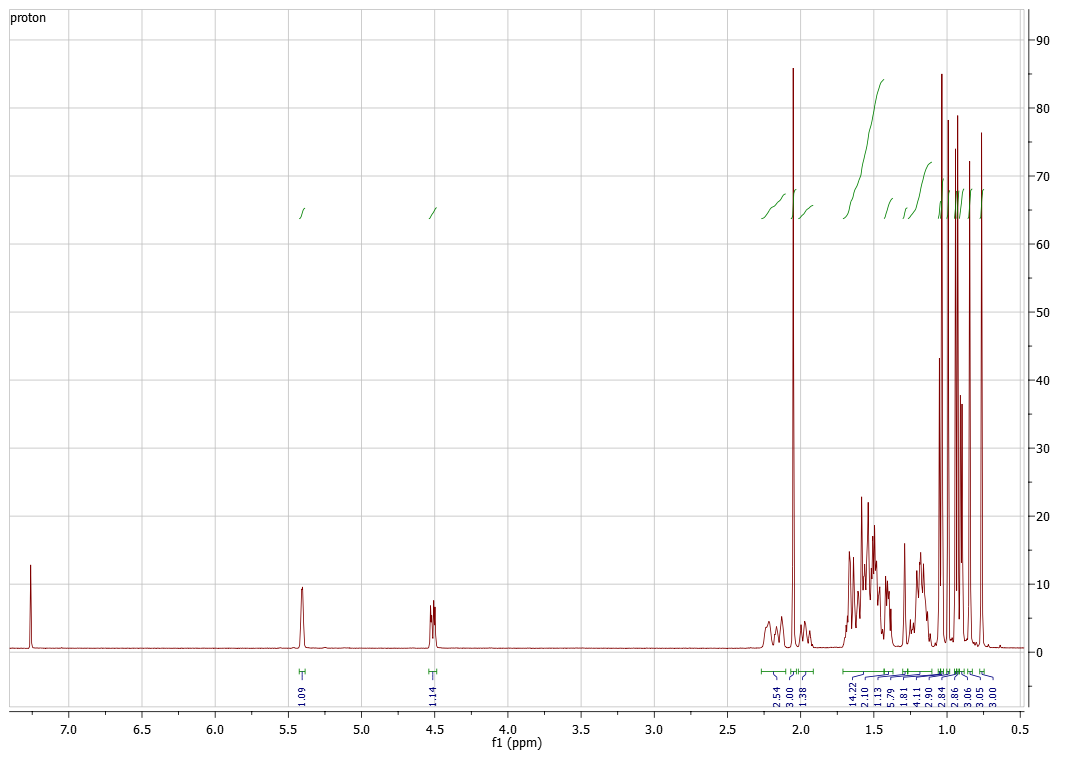


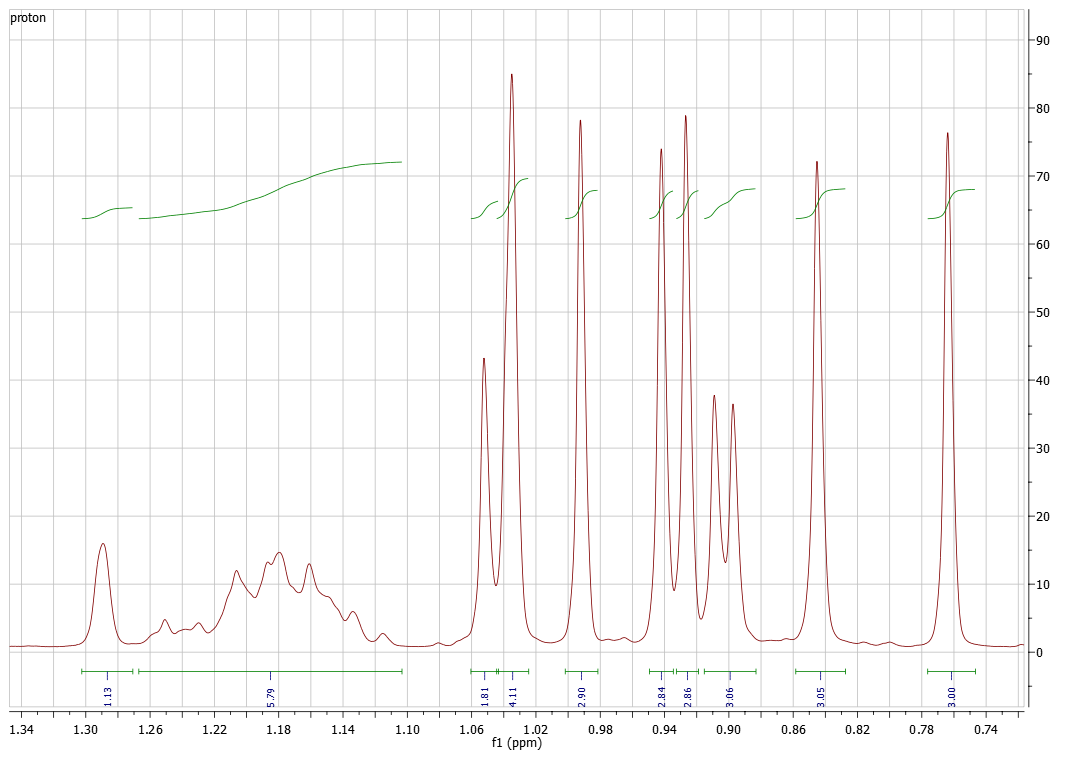


**Figure S1.** ^1^H NMR Data of **1**.


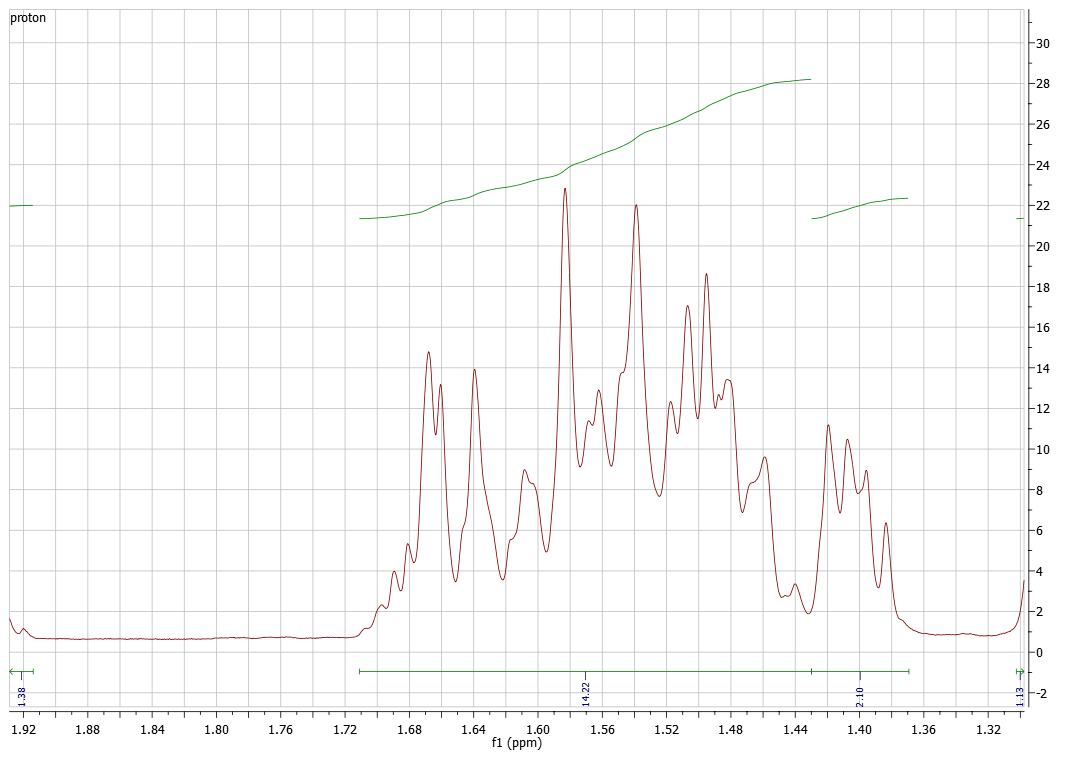


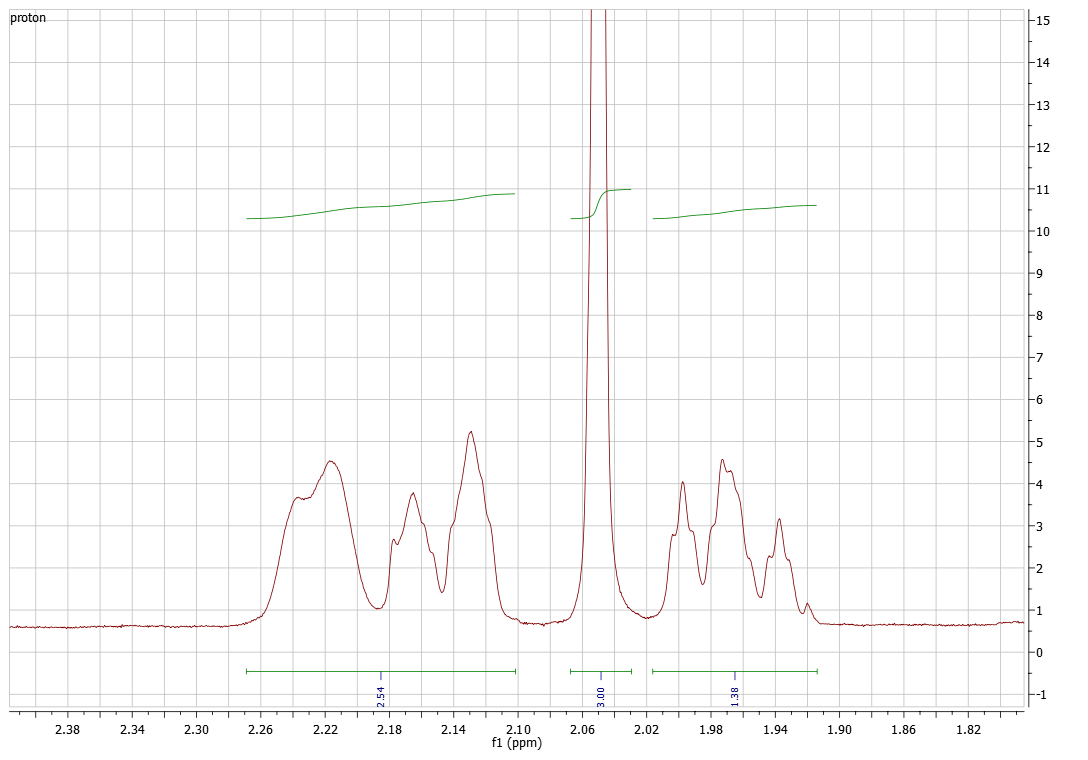


**Figure S1 Contd.** ^1^H NMR Data of **1**.


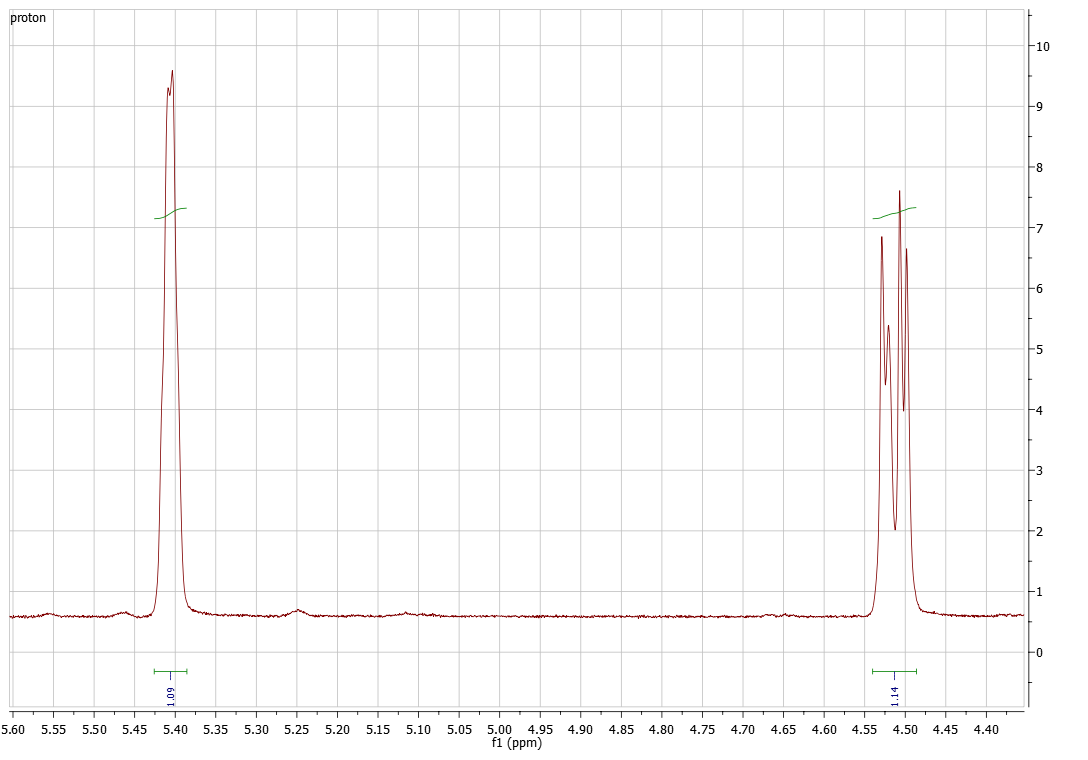


**Figure S1 Contd.** ^1^H NMR Data of **1**.


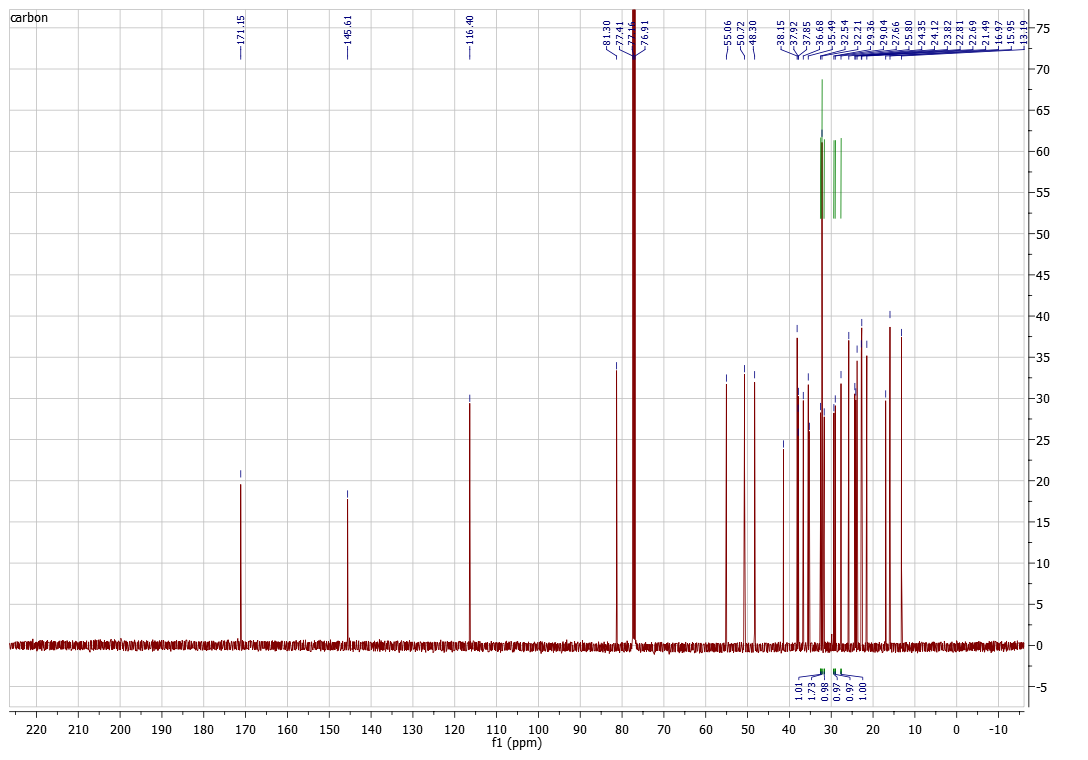


**Figure S1.** ^13^C NMR Data of **1**.


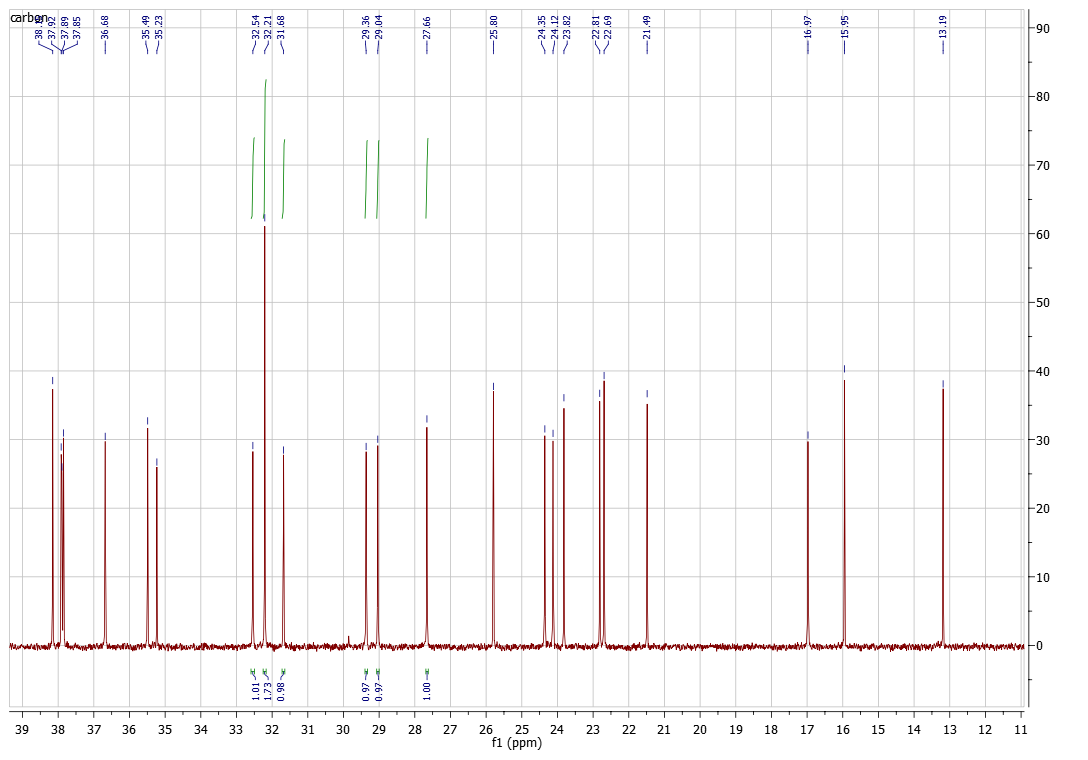


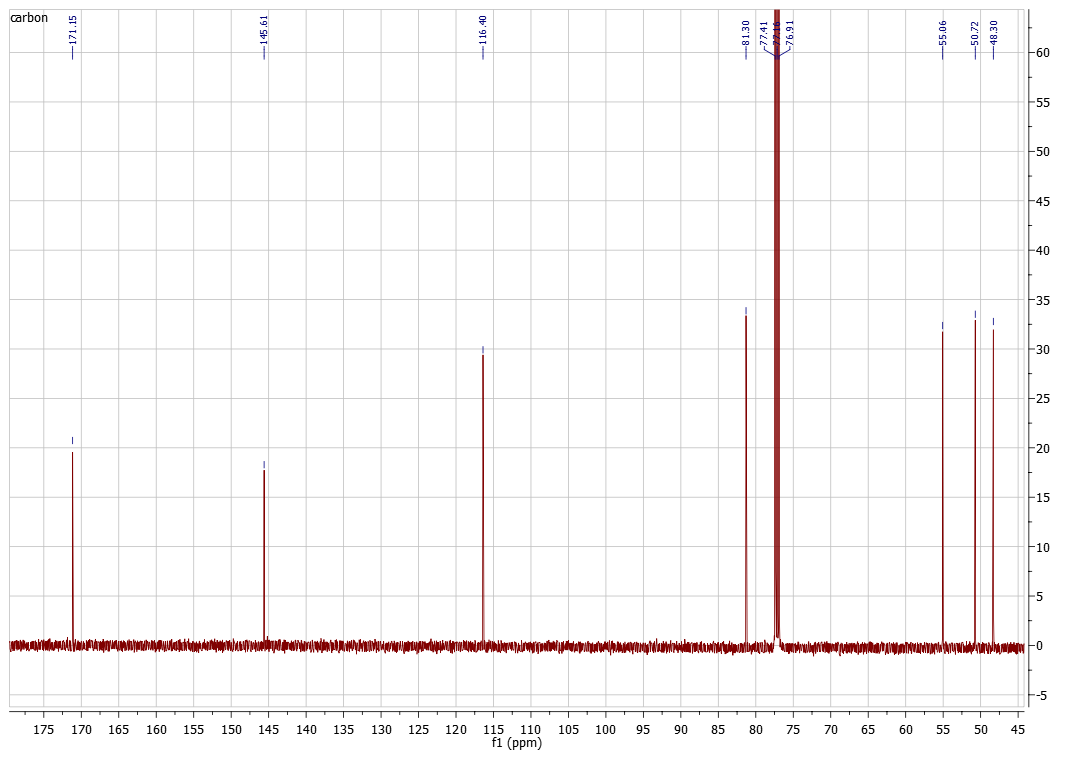


**Figure S1 Contd.** ^13^C NMR Data of **1**.


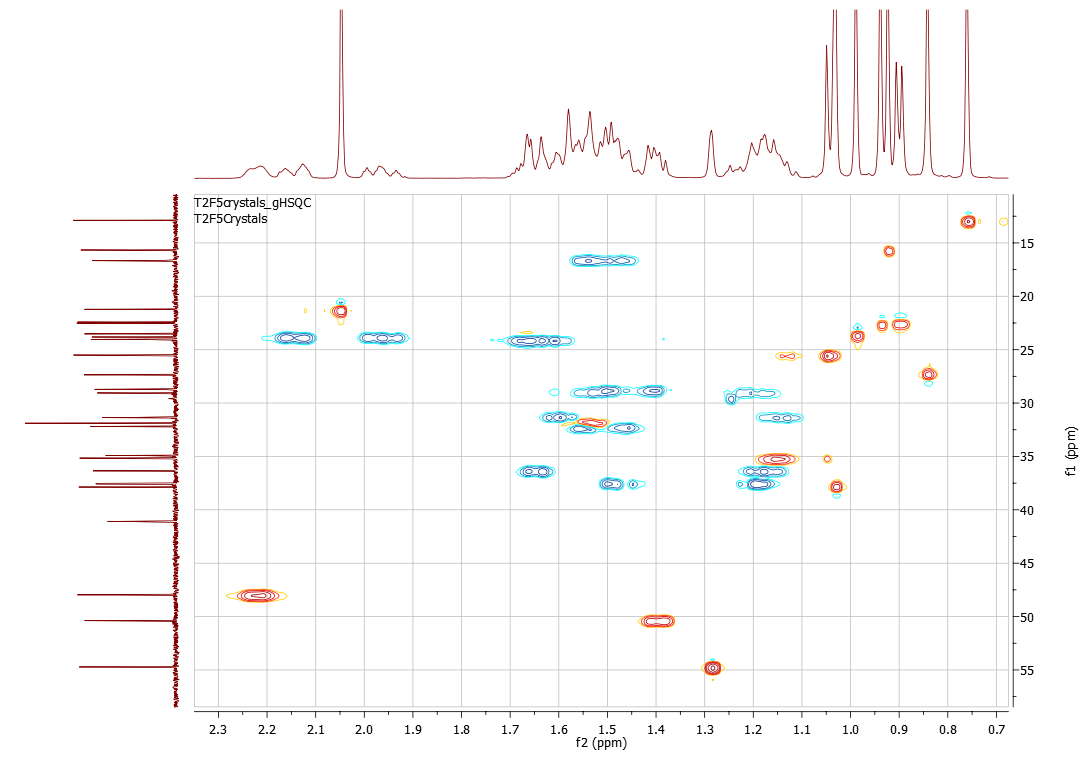


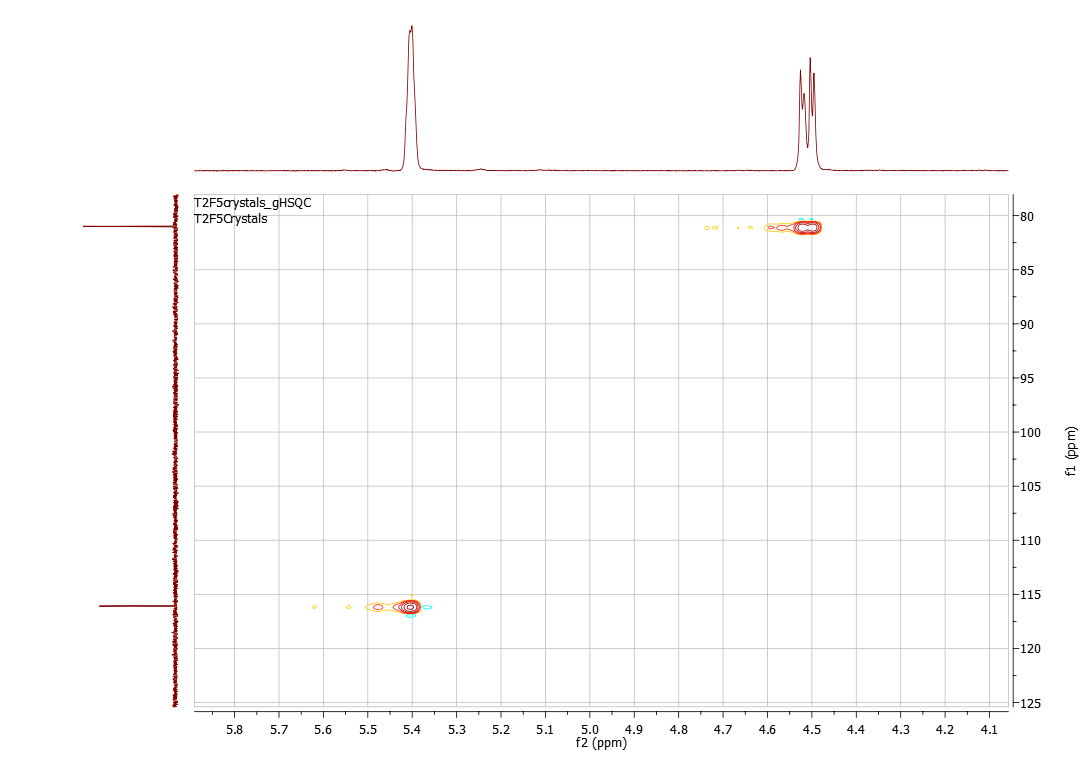


**Figure S1 Contd.** gHSQC NMR Data of **1**.


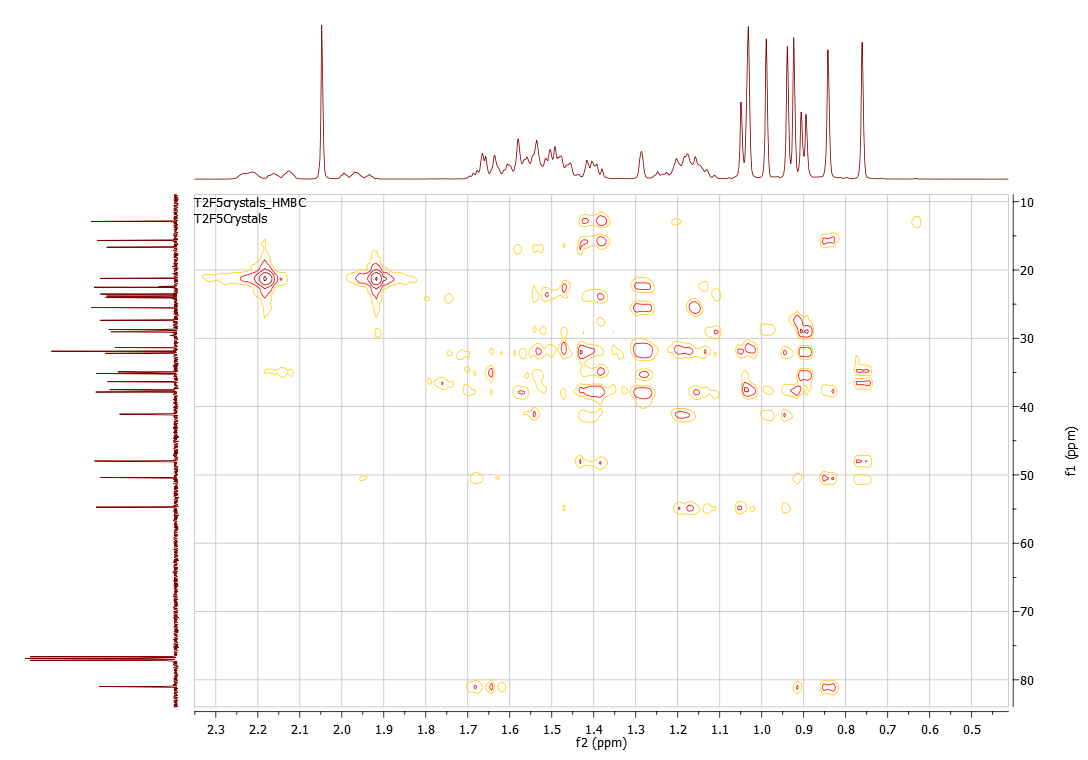


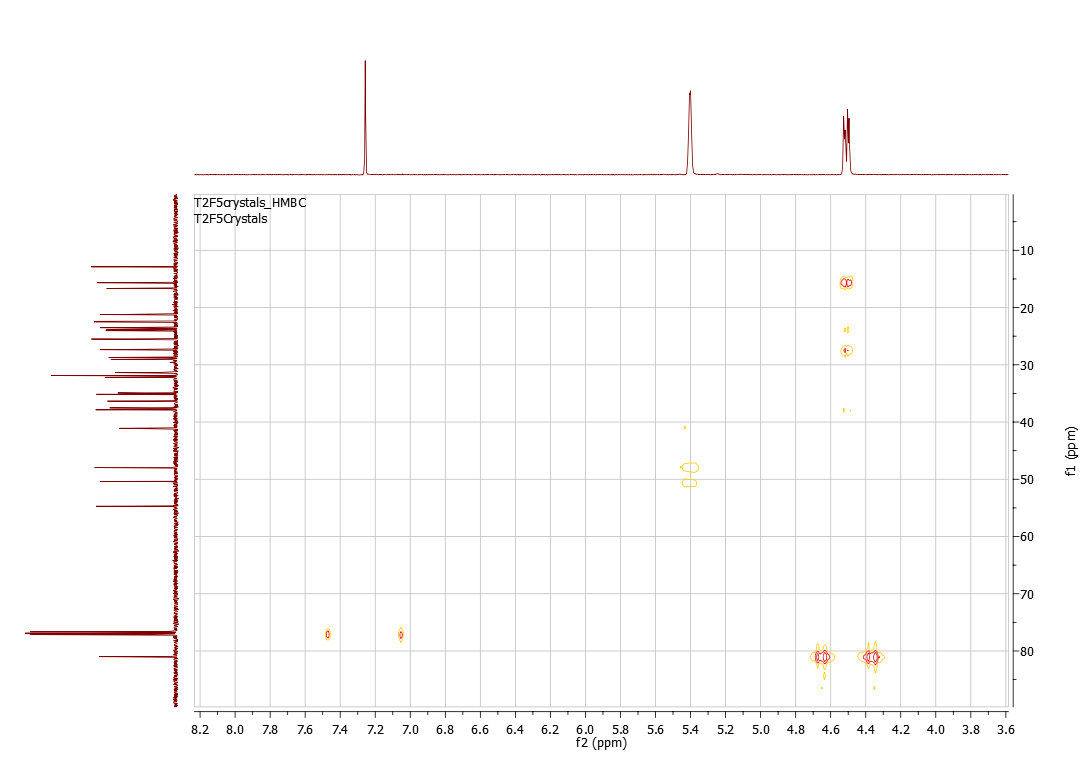


**Figure S1 Contd.** gHMBC NMR Data of 1.


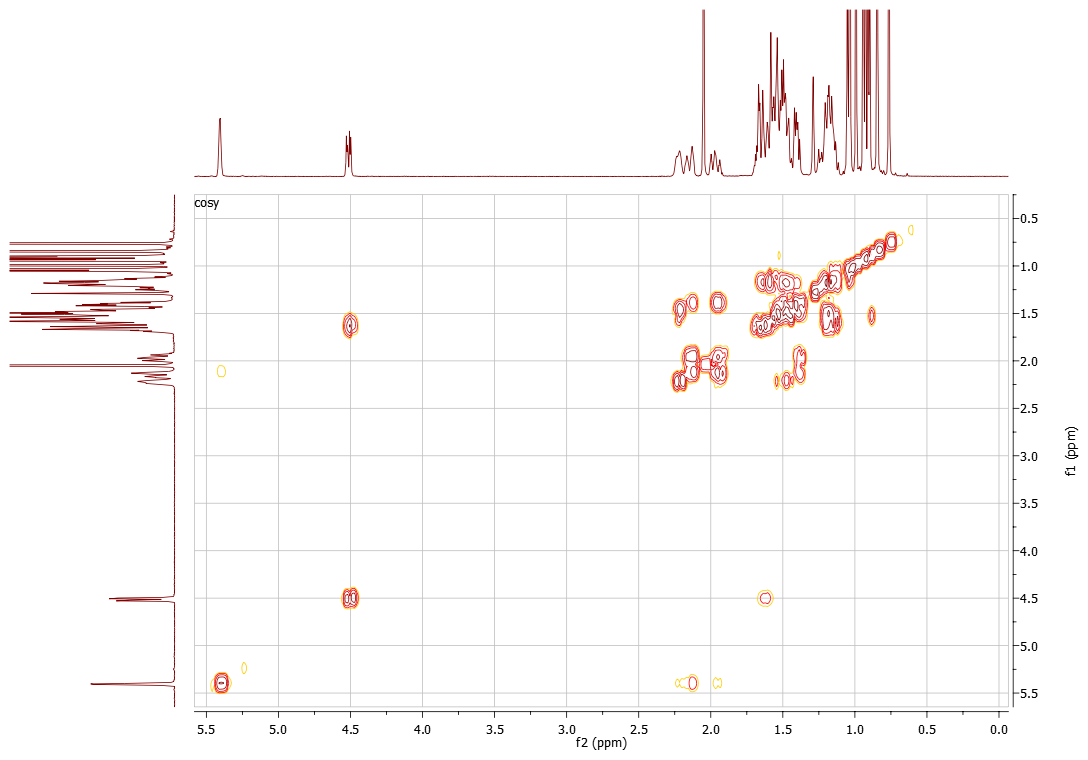


**Figure S1 Contd.** gCOSY NMR Data of **1**.


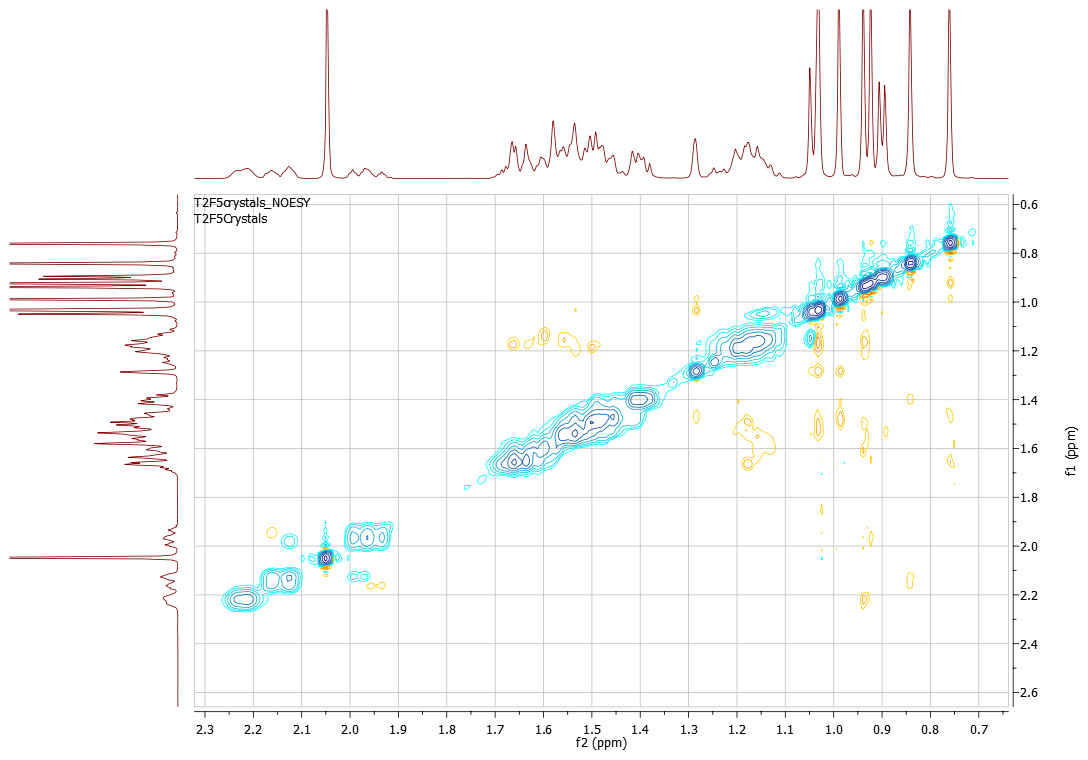


**Figure S1 Contd.** NOESY NMR Data of **1**.

**A**

**B**


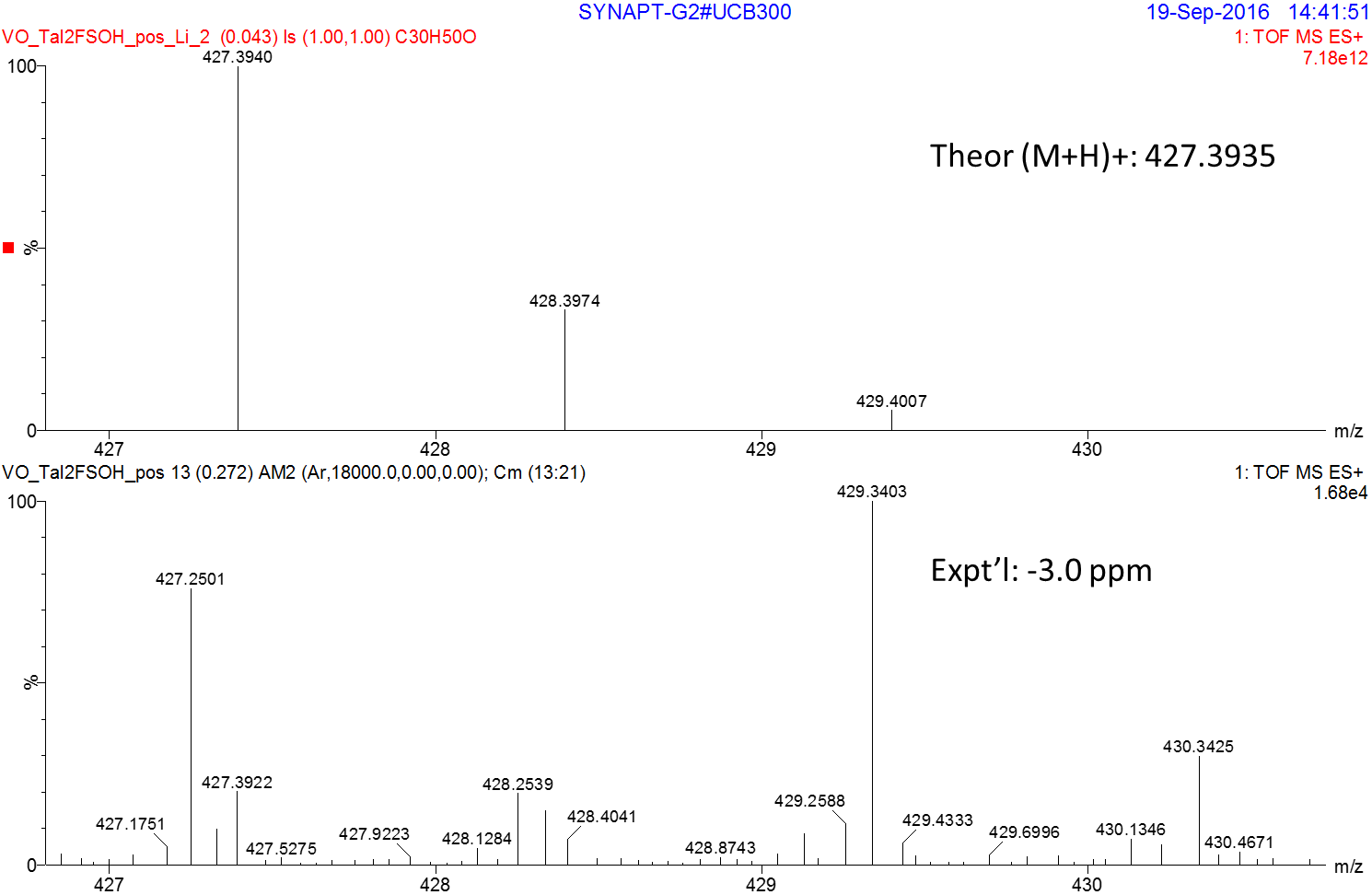


**Figure S2.** Mass Spectra Data of **1**(A) and **2**(B)

**Figure S3**. Levels of cholesterol in parasite treated with **2.** The peak height values are expressed in Log. The levels of cholesterol in the parasite was significantly increased after 4-hour exposure to 5 µM of **2**.


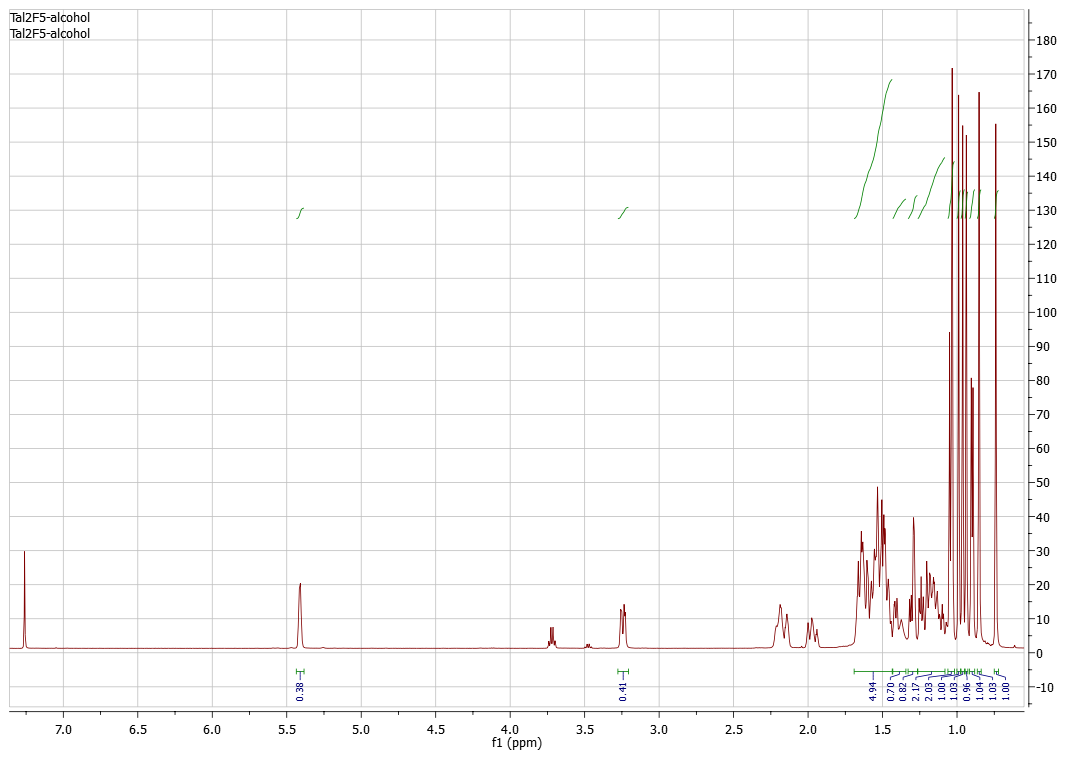


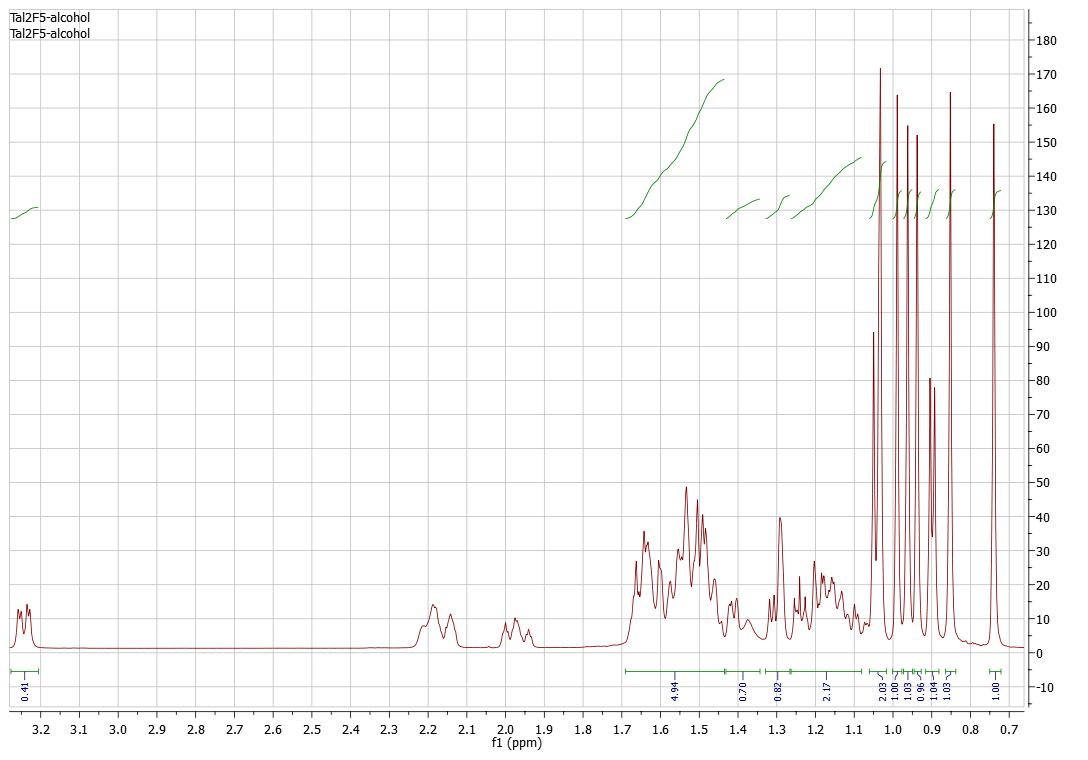


**Figure S4. ^1^**H NMR Data of **2**.


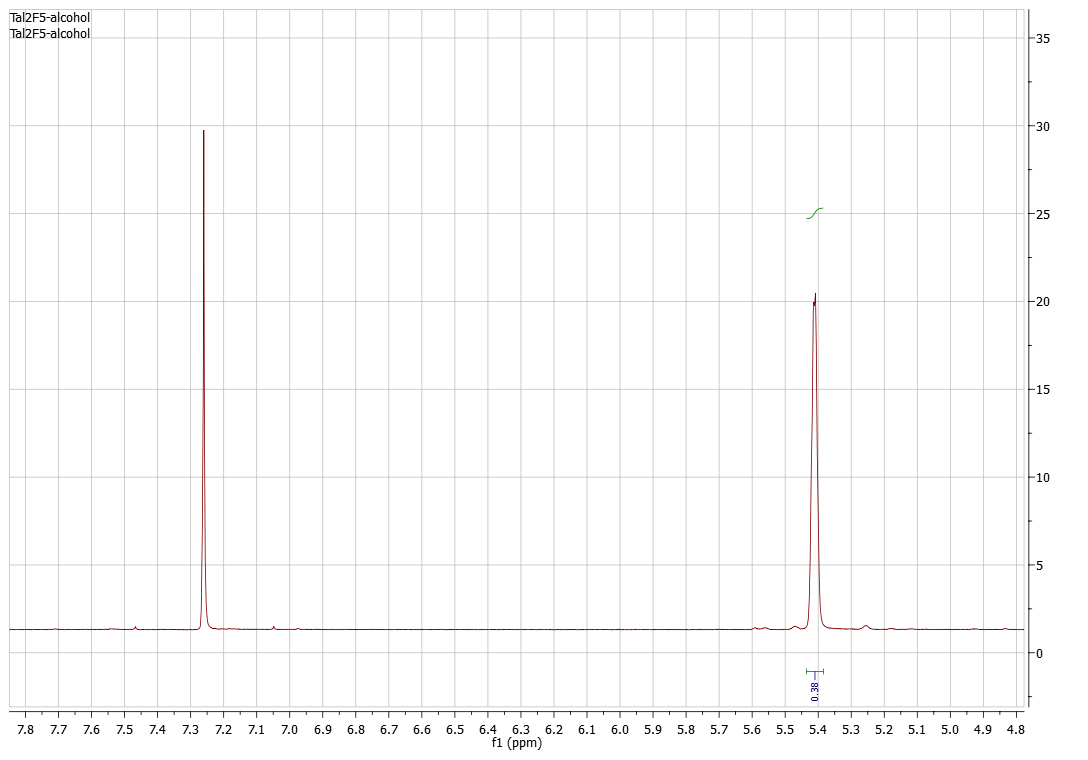


**Figure S4 Contd. ^1^**H NMR Data of **2**.


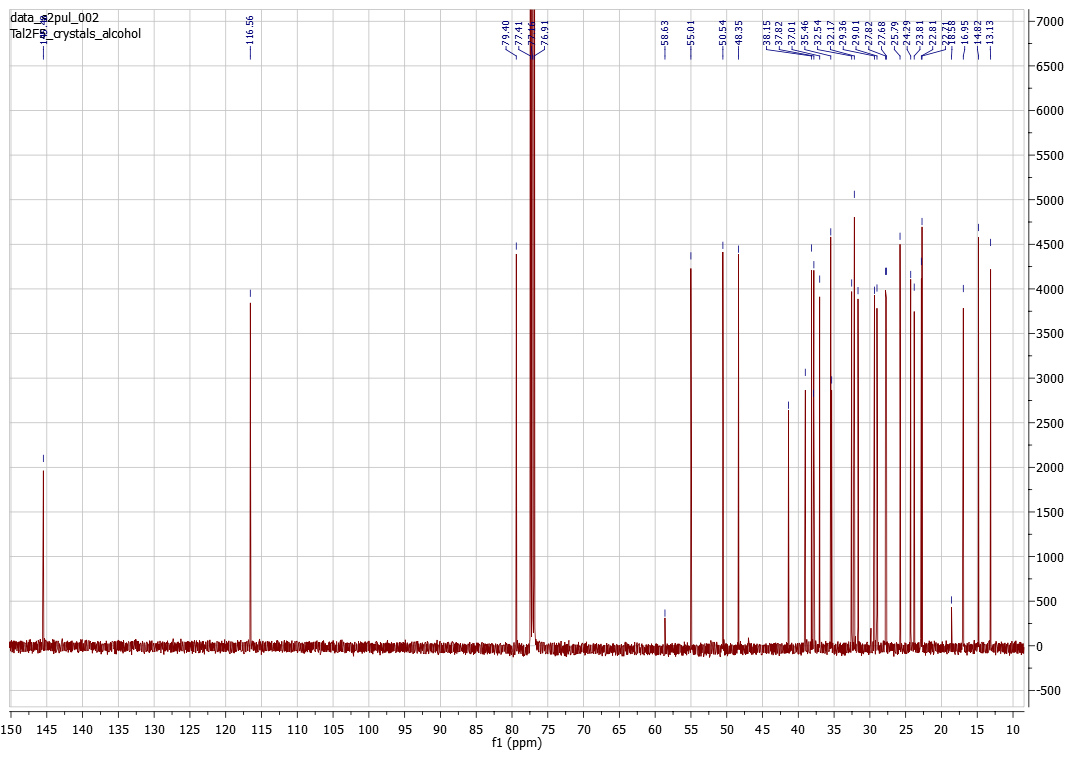


**Figure S4. ^13^**C NMR Data of **2**.


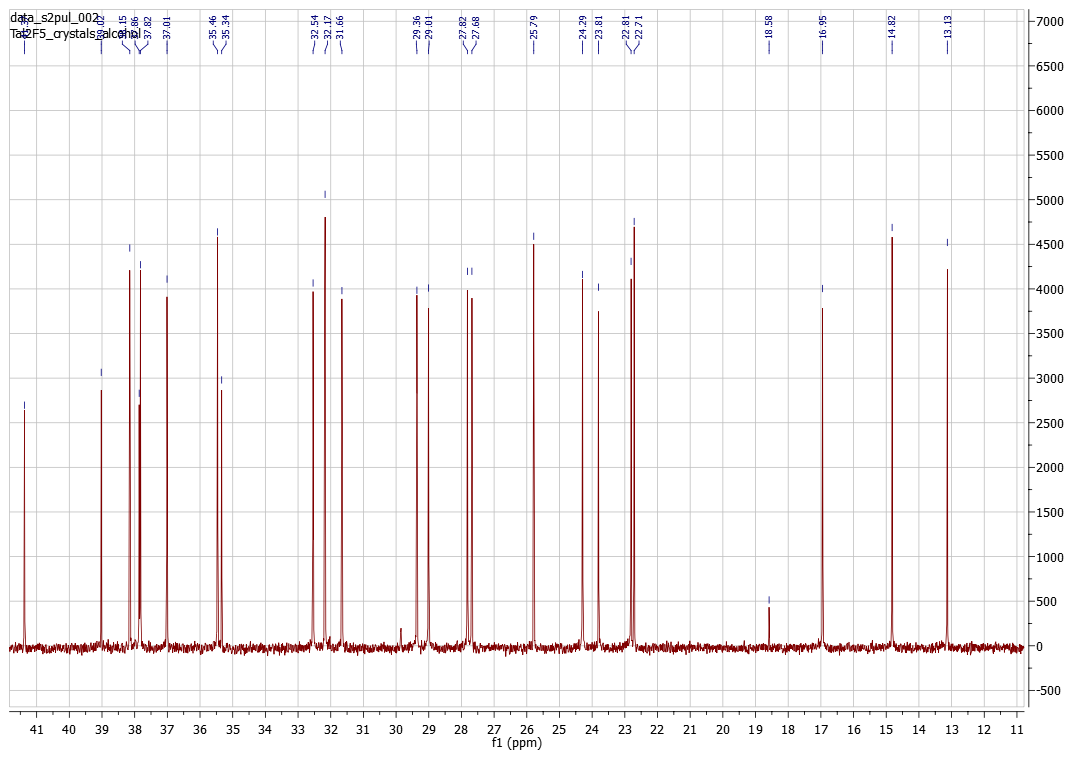


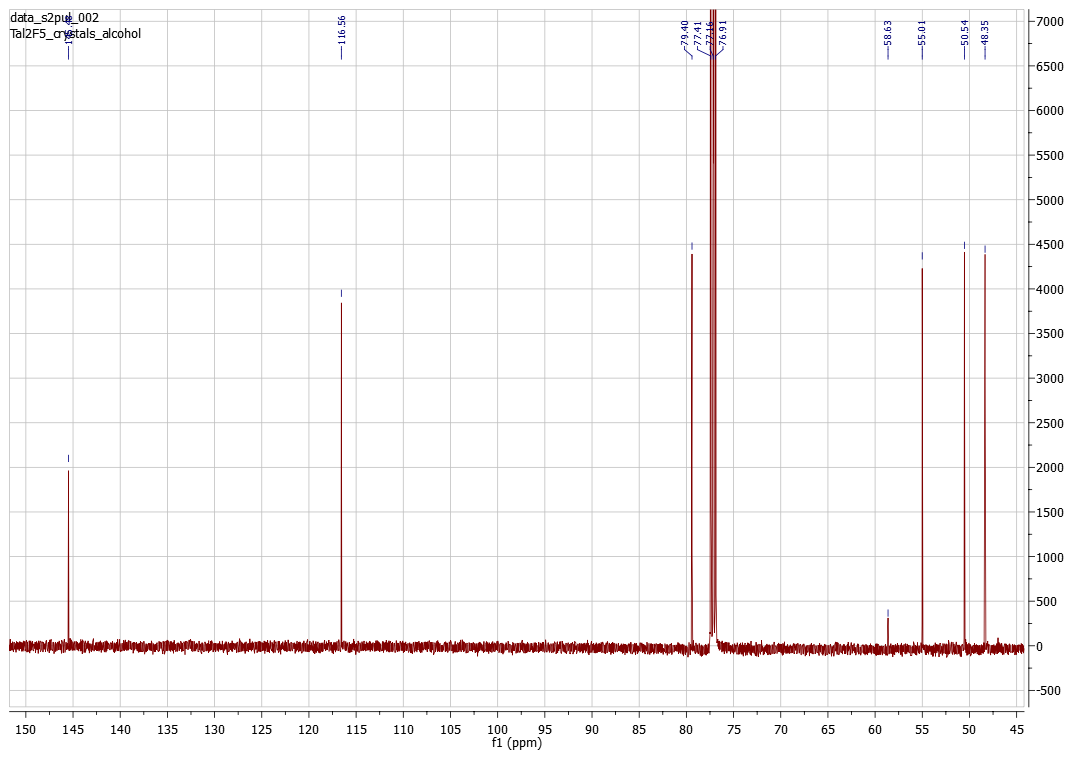


**Figure S4 Contd. ^13^**C NMR Data of **2**.


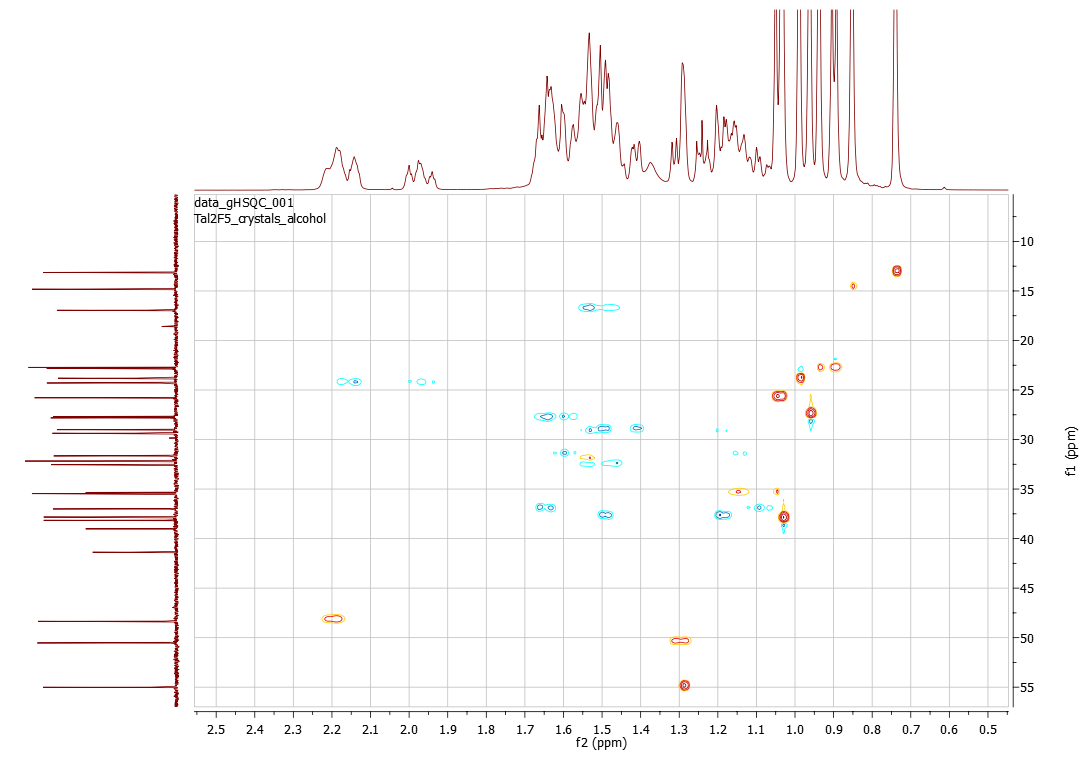


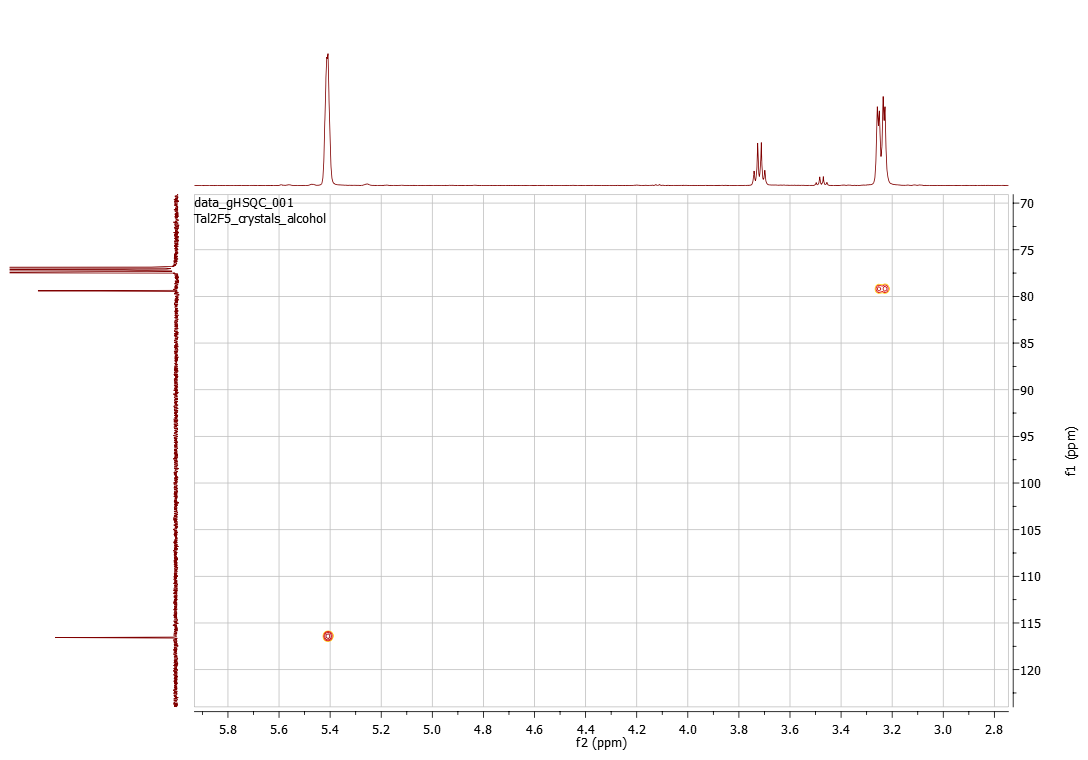


**Figure S4 Contd.** gHSQC NMR Data of **2**.


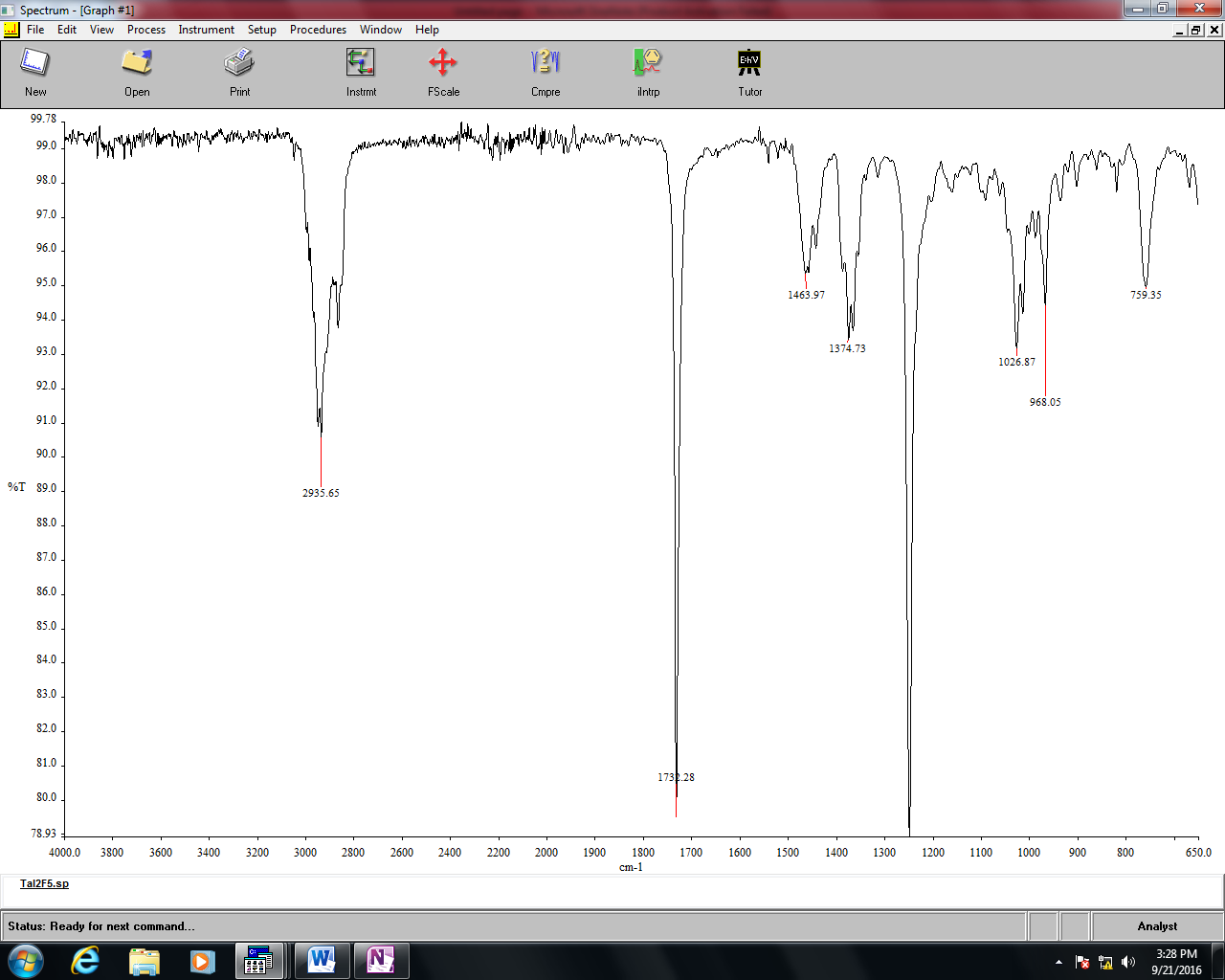


**A**

**B**

**Figure S5**. IR Spectra of **1**(A) and **2**(B).


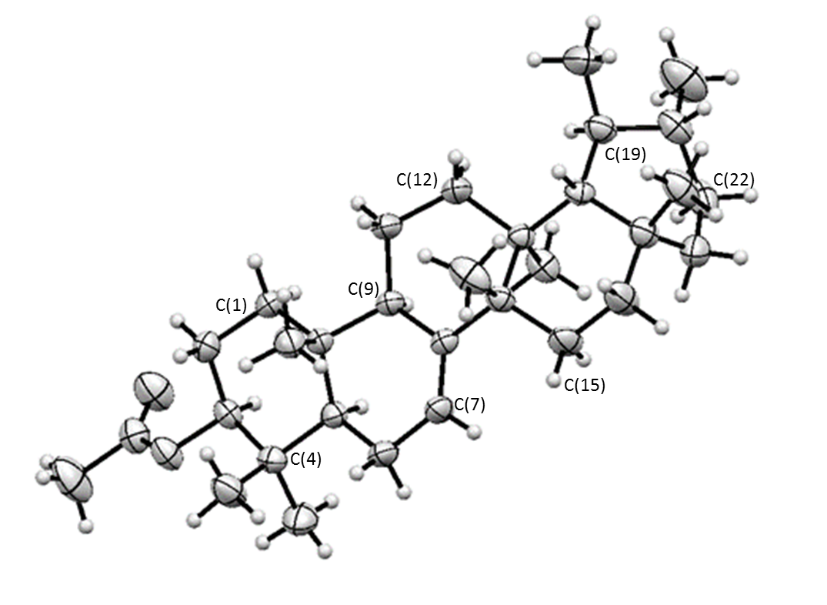


**Figure S6.** X-ray crystal structure (ORTEP drawing) of **1**


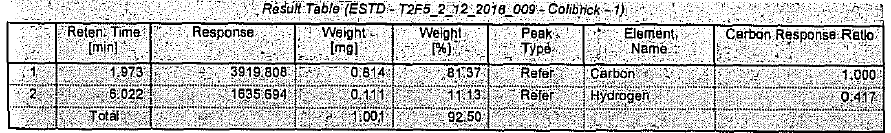


**Figure S7:** CH Elemental composition of **1**
